# Evolving perspectives on aortic stenosis: the increasing importance of evaluating the right ventricle before aortic valve intervention

**DOI:** 10.3389/fcvm.2024.1506993

**Published:** 2025-01-08

**Authors:** Vitaliy Androshchuk, Omar Chehab, Joshua Wilcox, Benedict McDonaugh, Natalie Montarello, Ronak Rajani, Bernard Prendergast, Tiffany Patterson, Simon Redwood

**Affiliations:** ^1^ School of Cardiovascular Medicine & Sciences, Faculty of Life Sciences & Medicine, King’s College London, London, United Kingdom; ^2^ Cardiovascular Directorate, St Thomas’ Hospital, London, United Kingdom; ^3^ School of Biomedical Engineering and Imaging Sciences, Faculty of Life Sciences & Medicine, King’s College London, London, United Kingdom; ^4^ Heart, Vascular & Thoracic Institute, Cleveland Clinic London, London, United Kingdom

**Keywords:** right ventricle, pulmonary hypertension, cardiovascular risk, aortic stenosis, aortic valve replacement

## Abstract

Aortic stenosis (AS) was historically considered a disease of the left side of the heart, with the main pathophysiological impact being predominantly on the left ventricle (LV). However, progressive pressure overload in AS can initiate a cascade of extra-valvular myocardial remodeling that could also precipitate maladaptive alterations in the structure and function of the right ventricle (RV). The haemodynamic and clinical importance of these changes in patients with AS have been largely underappreciated in the past. Contemporary data indicates that RV dilatation or impairment identifies the AS patients who are at increased risk of adverse clinical outcomes after aortic valve replacement (AVR). It is now increasingly recognised that effective quantitative assessment of the RV plays a key role in delineating the late clinical stage of AS, which could improve patient risk stratification. Despite the increasing emphasis on the pathological significance of RV changes in AS, it remains to be established if earlier detection of these changes can improve the timing for intervention. This review will summarise the features of normal RV physiology and the mechanisms responsible for RV impairment in AS. In addition, we will discuss the multimodality approach to the comprehensive assessment of RV size, function and mechanics in AS patients. Finally, we will review the emerging evidence reinforcing the negative impact of RV dysfunction on clinical outcomes in AS patients treated with AVR.

## 1 Introduction

Aortic stenosis (AS) is the most common valvular heart condition worldwide (1). The causes of AS show substantial geographic variation, with degenerative and age-related calcific aetiology being more common in Europe and North America, while rheumatic disease predominates as the most common cause in developing countries (2). The incidence of severe AS is up to 7% per year in patients >65 years old, with global estimates suggesting a progressive increase in prevalence due to population ageing (3, 4). According to conventional treatment planning principles, asymptomatic patients with significant AS and without any adverse prognostic features are considered to have a favourable prognosis with an annual mortality of ∼1% and generally follow a “watchful waiting” strategy (5). The onset of symptoms leads to a dramatic worsening of prognosis, with mortality rising to ∼95% at 5 years under conservative management, which should trigger prompt intervention in suitable patients (6). Transcatheter aortic valve implantation (TAVI) is now an increasingly well-established therapeutic option for severe AS in patients across the entire spectrum of operative risk (7). As a result, in many developed counties TAVI has surpassed surgical aortic valve replacement (SAVR) as the dominant invasive procedure for AS (8). When a bioprosthesis is required for the management of AS, the indications of TAVI vs. SAVR differ slightly between the European and North American guidelines. In the European guidelines, TAVI should be considered in patients >75 years old (9). In the North American guidelines, TAVI can be considered in patients >65 years old and TAVI is favoured in those >80 years old (10).

Although TAVI is technically feasible and can be offered to the majority of AS patients, around one third of high risk patients fail to derive a procedural benefit and remain severely symptomatic or die 1 year after treatment (11). As a result, refinement in patient selection for TAVI has been a subject of extensive research to accurately identify sub-groups of patients where intervention is associated with the best long-term clinical outcomes. Frailty, multiple co-morbidities and myocardial remodeling are now recognised as important prognosis markers in TAVI (12). Of these factors, improvements in screening and comprehensive assessment of extra-valvular myocardial remodeling for risk prediction has gained increased attention. Indeed, the benefits of intervention may be limited by the development of advanced downstream myocardial changes with progressive AS. Likewise, early signs of remodeling in patients with asymptomatic severe and moderate AS could identify patients who may benefit from an early AVR. Integrating these changes into AS assessment may refine traditional AS classification and help to guide the optimal timing for intervention.

Current recommendations driving the decision-making process in the treatment of AS are based mainly on 3 parameters: (1) the haemodynamic severity of AS, defined using the transvalvular gradients and aortic valve area, (2) the presence of symptoms and (3) the left ventricular (LV) ejection fraction (9, 10). This reflects the early investigations into cardiac remodeling abnormalities as predictors of poor outcomes in AS, which focused primarily on left-sided cardiac structures, particularly the LV. In comparison, the impact of AS and its progression on the right ventricle (RV) has traditionally received little attention. The reasons for this are historically attributed to the complex RV geometry that can be challenging to assess using standard imaging techniques, the lack of consistent methodology to define RV dysfunction and because the contribution of the RV to the overall cardiac haemodynamics was unclear. In this context, the RV has often been referred to as the forgotten ventricle and considered little more than a passive conduit that passes the blood to the pulmonary circulation. However, this misconception was first defied when increased mortality was associated with RV disease states, including RV infarction, atrial septal defect and cor pulmonale (13). Since then, the importance of RV function for risk stratification and prediction of clinical outcomes has been increasingly recognised in a wider variety of cardiovascular and pulmonary diseases, including patients with AS (14). RV dysfunction is common in AS and varies in prevalence according to the stage of the disease, ranging from ∼25% in normal-flow high-gradient AS with preserved LV ejection fraction (LVEF) to ∼55% in low-flow low-gradient AS and reduced LVEF (15, 16). The novel system for anatomical and functional classification of the patients with severe AS, which takes into account the function of the RV in addition to the LV, has now been extensively validated (17). Despite this, the current guidelines do not make any specific recommendations about the role of RV function in AS management. Furthermore, neither the surgical nor TAVI-specific mortality prediction models include any variables related to RV function or morphology (18). The objective of this review is to increase awareness of RV pathology in AS patients by focusing on the current understanding of RV remodeling pathophysiology in AS, the main imaging modalities for assessing RV function in AS, the prognostic impact of RV dysfunction in AVR patients and the knowledge gaps that exist in this area.

## 2 Right ventricular physiology under normal conditions

**Figure 1:**
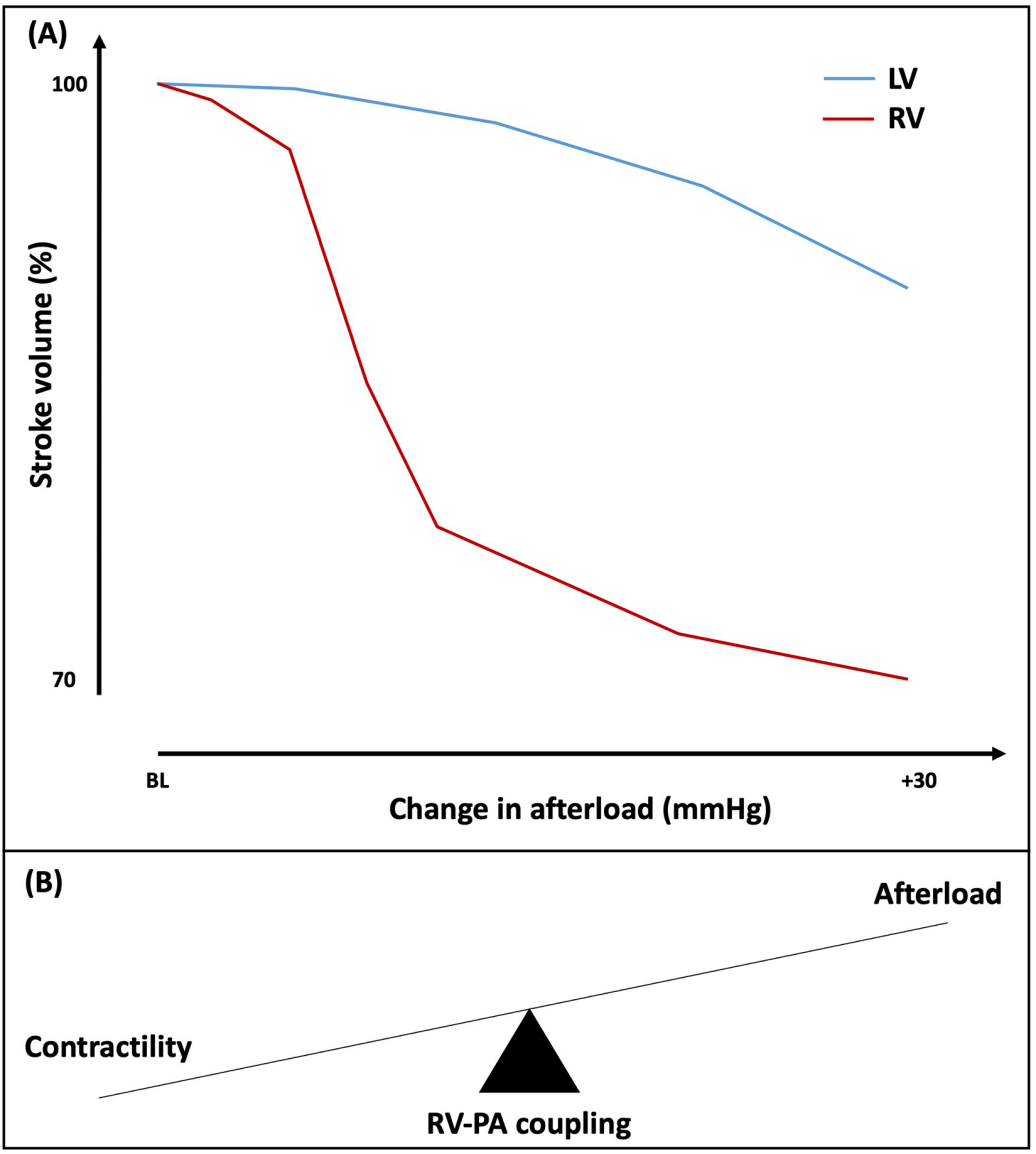
**(A)** Effect of increasing afterload on right and left ventricular stroke volume. **(B)** Schematic representation of right ventricular-pulmonary artery (RV-PA) coupling. BL, baseline; LV, left ventricle; RV, right ventricle; PA, pulmonary artery.

The structure and physiology of the RV is fundamentally different to the LV. The RV is a thin-walled and high-volume structure, with myofibers arranged longitudinally in the deep subendocardial layer and circumferentially in the superficial subepicardial layer, which is continuous with the LV (19). Overall RV function is a reflection of intrinsic contractility, pre-load, after-load, constraint within the pericardium and interaction with the LV. Under normal pre-load and after-load conditions, the RV ejects the same stroke volume (SV) of blood as the LV but with approximately 25% of the stroke work (20). Most of the SV is generated predominantly through the longitudinally oriented myofibers. The RV is usually coupled with low-impedance, low-pressure and highly distensible pulmonary vasculature. The RV is more compliant than the LV and has substantial SV reserve, enabling it to effectively accommodate an increase in volume load with minimal increases in pressure (21). On the contrary, the RV tolerance to increased pressure loads is much lower, which can bring about a marked reduction in SV (Figure 1A) (22). As a result, there has been increasing appreciation for the importance of ventricular-arterial interaction for the right heart, with some authors suggesting that the RV and pulmonary circulation are best viewed as a combined cardiopulmonary functioning unit (23). This is analogous to valvulo-arterial impedance (Zva) on the left side of the heart, which estimates global LV afterload imposed by AS and reduced arterial compliance, and improves prediction of mortality in patients with AS (24). The RV ability to offset the afterload and transfer the energy efficiently from the right heart to the pulmonary vessels largely depends on the satisfactory coupling between the RV and the pulmonary circulation, known as RV to pulmonary artery (RV-PA) coupling (Figure 1B) (25). The concept of coupling is particularly important in physiologically describing the continuum of ventricular adaptations to increasing pulmonary arterial pressures.

## 3 Pathophysiology of right ventricular dysfunction in aortic stenosis

**Figure 2:**
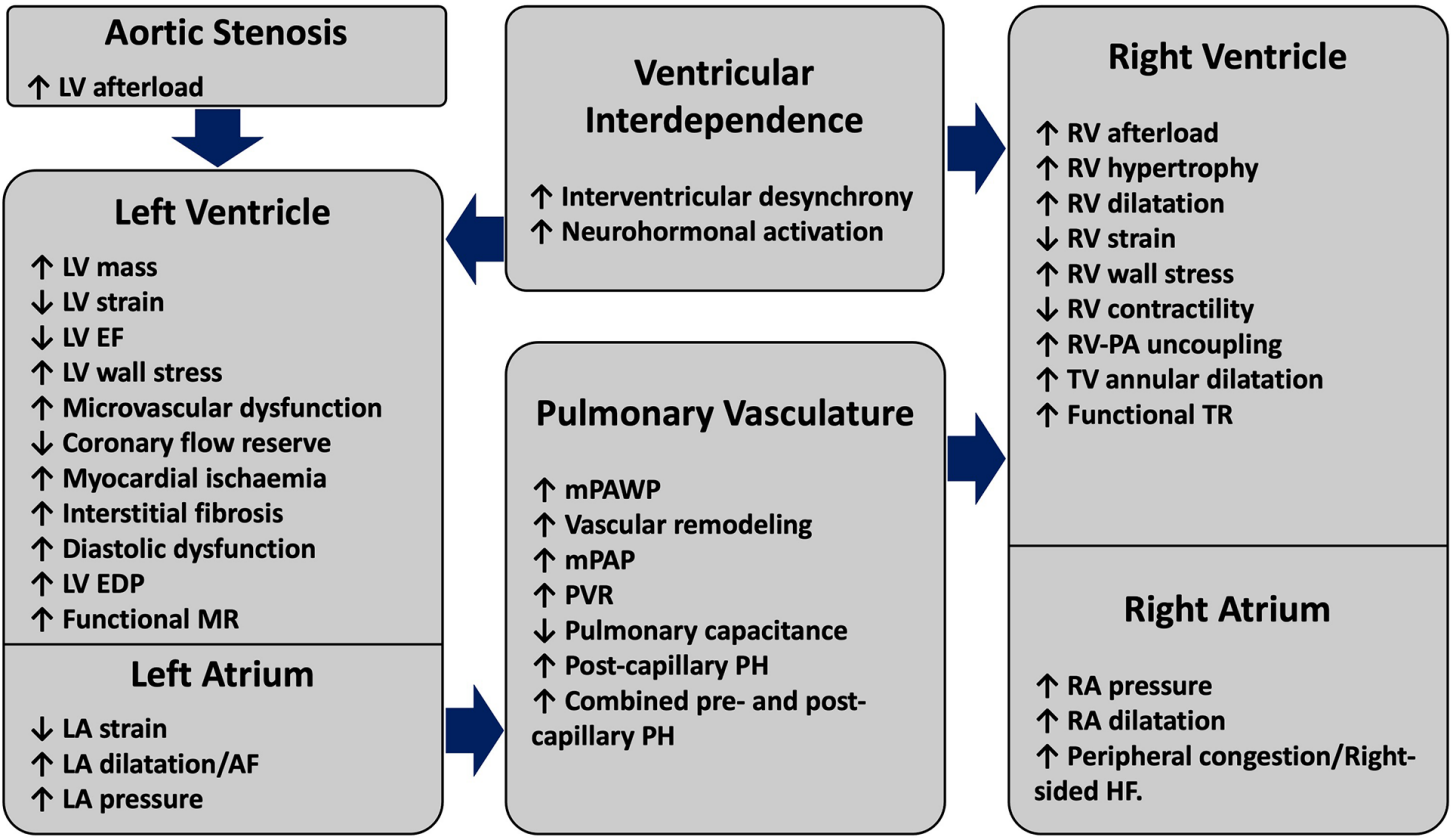
Structural and functional alterations driving the pathophysiology of right ventricular failure in aortic stenosis. AS-induced pressure overload initiates a cascade of compensatory mechanisms, which initially produce maladaptive alterations effecting the LV and the LA. With disease progression and persistently elevated left-sided heart pressures, pulmonary vascular changes precipitate the onset of PH. As a result of PH, progressive RV-PA uncoupling and RV dysfunction can occur, with the onset of RV failure being marked by symptoms related to elevated right-sided filling pressures. Pathological LV and RV interdependence can exacerbate biventricular dysfunction. AS, aortic stenosis; EDP, end-diastolic pressure; EF, ejection fraction; LV, left ventricle; mPAP, mean pulmonary artery pressure; MR, mitral regurgitation; RV, right ventricle, PA, pulmonary artery; PVR, pulmonary vascular resistance; mPAWP, mean pulmonary artery wedge pressure; PH, pulmonary hypertension; TR, tricuspid regurgitation; TV, tricuspid valve.

The pathophysiology of RV dysfunction as a consequence of AS is schematically summarised in Figure 2. The myocardial damage in severe AS frequently extends beyond the aortic valve to create a spectrum of extra-valvular alterations in cardio-pulmonary structure and function. The development of RV impairment in long-standing AS typically indicates an advanced disease state with exhausted maladaptive changes of the LV. Increased valvular resistance in progressive AS causes LV pressure overload and initially leads to a compensatory increase in LV wall thickness to normalise the systolic wall stress (26). However, LV hypertrophy is associated with a cascade of maladaptive remodeling changes, which ultimately lead to diminished LV performance. A combination of increased myocardial oxygen demand, reduced coronary flow reserve and microvascular dysfunction lead to subendocardial ischaemia and interstitial fibrosis, thereby potentiating increased LV stiffness and impaired relaxation (27, 28). These alterations result in worsening diastolic dysfunction, which is described by a rise in LV end-diastolic pressure (LVEDP) for a normal LV end-diastolic volume (LVEDV). This is reflected in a shift of LV end-diastolic pressure volume relationship upwards and to the left (29). Worsening diastolic dysfunction and increased LVEDP lead to a further reduction in coronary perfusion pressure, which increases ischemia and perpetuates fibrosis (30). Various phenotypes of LV systolic dysfunction can co-exist with these pathological changes, ranging from a reduction in strain on echocardiography with preserved LVEF to frank LV systolic impairment (31). With disease progression, left atrial (LA) dysfunction and/or functional mitral regurgitation (MR) can occur (32). The development of atrial fibrillation is associated with worse haemodynamic profile compared to patients in sinus rhythm and likely plays an important role in PH progression (33). Collectively, these alterations result in an elevated LA pressure as a compensatory response to counteract the increased resistance to LV filling (34). As a consequence of backwards pressure transmission, mean pulmonary capillary wedge pressure (mPCWP) increases, marking the development of isolated post-capillary pulmonary hypertension (Ipc-PH). Through poorly understood mechanisms involving endothelial dysfunction, neurohormonal activation and the release of pro-inflammatory cytokines, chronic and significant elevation of pulmonary artery pressures can result in global pulmonary vascular remodeling and intimal thickening (35). These abnormalities increase pulmonary vascular resistance (PVR), reduce pulmonary arterial capacitance and increased RV systolic and diastolic pressures, which can progress to combined pre and post-capillary PH (CpcPH) (36). Right heart catheterization identifies PH in ∼50%–75% patients with AS undergoing AVR, with CpcPH seen in ∼20%–25% of these patients (37). Reflecting the more advanced disease stage, CpcPH is associated with higher mPCWP, lower pulmonary arterial capacitance and increased mortality compared to other PH groups (36, 38). Although time to disease progression and the onset of more advanced PH remains unclear, higher level of N-terminal-proBNP (NT-proBNP) has been proposed to predict CpcPH and increased post-AVR mortality (39).

**Figure 3:**
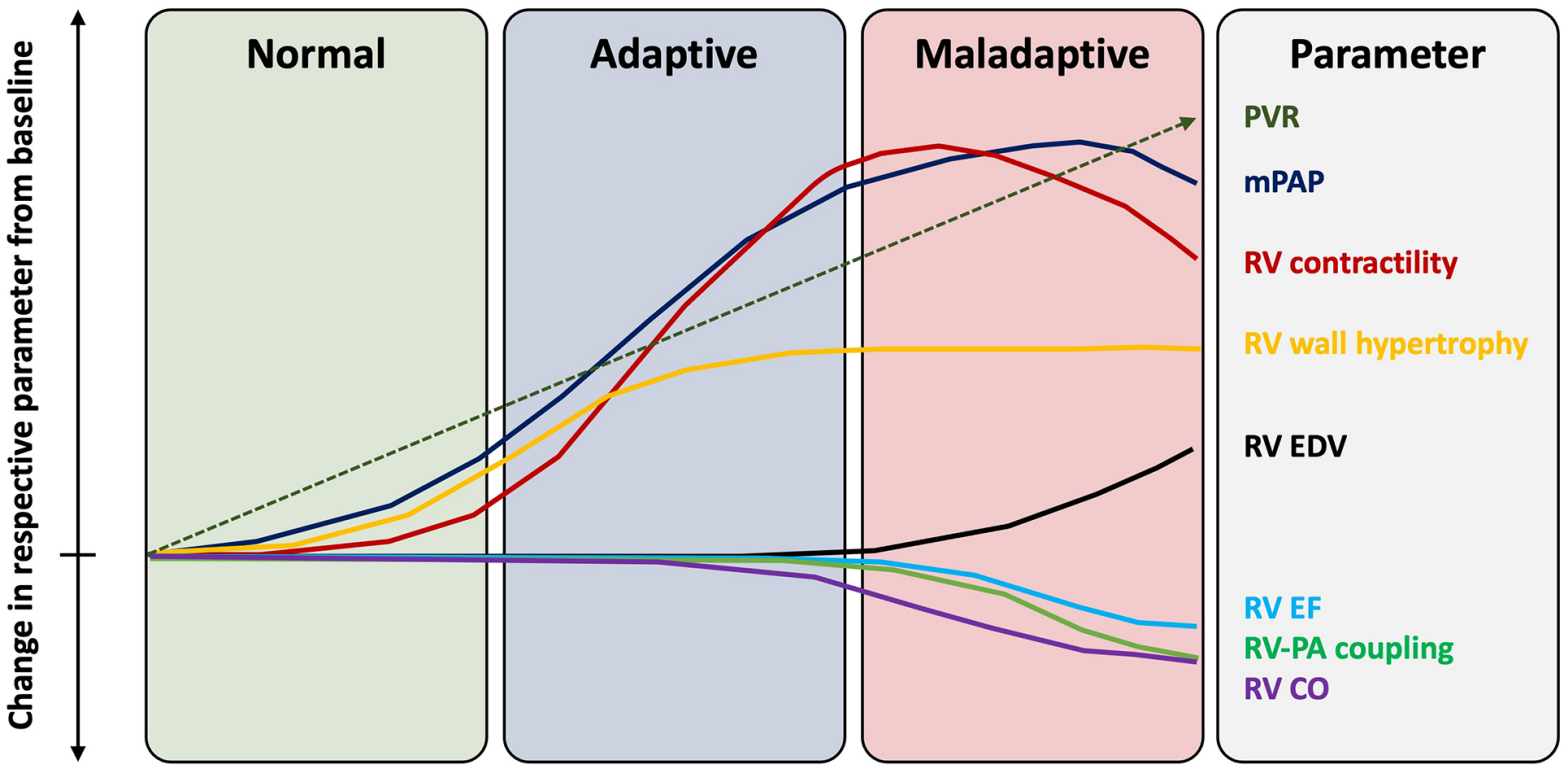
Changes in right ventricular function with progressive pulmonary hypertension. EF, ejection fraction; mPAP, mean pulmonary artery pressure; PA, pulmonary artery; PVR, pulmonary vascular resistance; RV, right ventricle.

In the absence of primary lung pathology, RV dysfunction in AS represents an advanced stage of cardiac injury, which manifests as the sum of progressive “downstream” structural and functional abnormalities affecting the LV, LA, mitral valve and pulmonary circulation. The RV adaptation to PH represents a continuum with initial compensatory mechanisms at one end and a maladaptive changes on the other. In the early stages, the RV can appropriately accommodate increased afterload with adaptive concentric hypertrophy, which increases contractility 4/5-fold and preserves the SV (40). However, progressive and sustained PH in advanced AS can lead to pathological RV remodeling. This includes initial RV dilatation, which develops in an attempt to maintain SV and CO via the Frank-Starling mechanism (41). Adaptive increase in RV mass and subsequently RV dilatation increase myocardial wall stress and oxygen consumption, which promotes ischaemia, fibrosis and stiffness (42). Functional tricuspid regurgitation (TR) can also develop as a consequence of RV and tricuspid valve annular dilatation, causing RV volume loading and further dilatation. Additionally, PH can shift the interventricular septum (IVS) towards the LV in systole, which potentiates interventricular desynchrony, RV mechanical inefficiency, LV underfilling and myocardial atrophy (43). RV-PA uncoupling occurs when sustained increases in pulmonary load cannot be matched with compensatory RV adaptations. Sustained increase in RV afterload can eventually exhaust the RV adaptive abilities, resulting in RV contractile dysfunction and a reduction in CO (Figure 3). RV failure occurs when it can no longer support blood flow in the circulation and accommodate the venous return without an increase in right atrial filling pressure, which leads to systemic venous congestion (44).

Increased afterload caused by PH is considered the main determinant of RV dysfunction in left-sided heard diseases such as AS (45). However, RV dysfunction is not always a stepwise phenomenon since RV behaviour can also be modulated through ventricular interdependence in pressure-loaded LVs (43). Ventricular interdependence refers to the concept where AS-induced alterations in LV configuration and function can be transmitted to cause RV dysfunction through direct mechanical interactions between the ventricles and independently of circulatory connections. Serial interactions occur because the RV and LV pump through the pulmonary and systemic circulations in series. Parallel interactions are mechanically plausible because the RV and LV are constrained within the same pericardial space and also intricately connected at the IVS, with shared common fibres that encircle both ventricles. The role of ventricular interdependence in mediating RV dysfunction is supported by several experimental models and clinical studies of AS, which showed a positive correlation between increased LV afterload and RV free wall remodeling on cardiac magnetic resonance imaging (CMR) (46, 47). Furthermore, RV functional parameters show a strong correlation with LVEF, LV global longitudinal strain and mean aortic gradient in severe AS, whereas the correlation between RV function and pulmonary artery systolic pressure (PASP) is much weaker (15). Some authors have also postulated that molecular pathways mediated by the growth-stimulating signals like angiotensin-1 and catecholamines produced in the hypertrophied LV may contribute to RV remodeling in AS (48).

## 4 Assessment of right ventricular function in aortic stenosis

**Figure 4:**
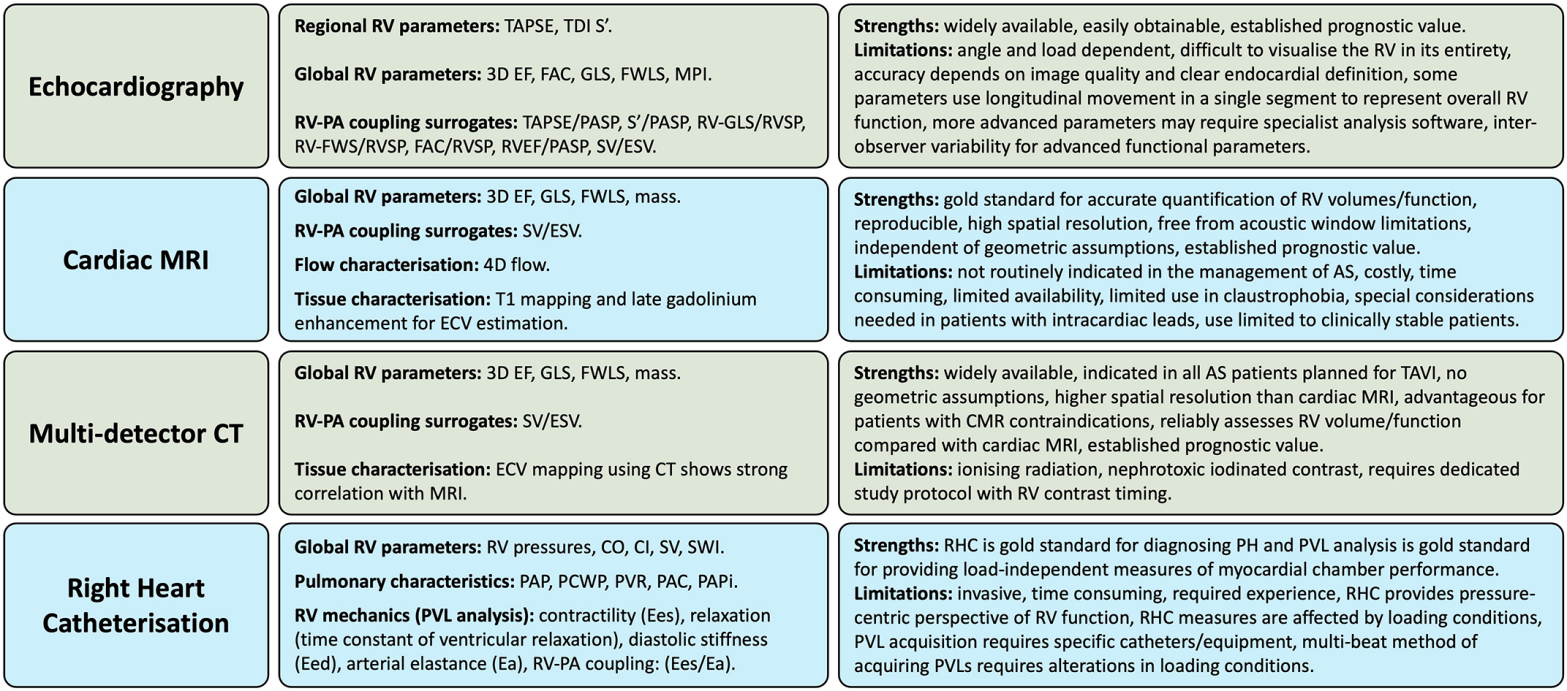
Multi-modality approach to assessing right ventricular function in aortic stenosis. AS, aortic stenosis; CO, cardiac output; CI, cardiac index; CT, computed tomography; Ea, arterial elastance; Eed, end-diastolic elastance; Ees, end-systolic elastance; EF, ejection fraction; ESV, end-systolic volume; FAC, fractional area change; FWLS, free-wall longitudinal strain; GLS, global longitudinal strain; mPAP, mean pulmonary artery pressure; MPI, myocardial performance index; MRI, magnetic resonance imaging; RV, right ventricle, PA, pulmonary artery; PCWP, pulmonary capillary wedge pressure; PAC, pulmonary arterial compliance; PAPi, pulmonary arterial pulsatility index; PAP, pulmonary artery pressure; PASP, pulmonary artery systolic pressure; PH, pulmonary hypertension; PVL, pressure-volume loop; PVR, pulmonary vascular resistance; RHC, right heart catheterisation; RVSP, right ventricular systolic pressure; SV, stroke volume; SWI, stroke work index; TAPSE, tricuspid annular plane systolic excursion; TDI, tissue doppler imaging; 3D, 3-dimentional.

Given the complex RV geometry and interplay between preload, afterload and contractility as the determinants of its mechanical performance, assessment of RV function is best performed using a multimodality approach. The ideal imaging technique should allow comprehensive, accurate and reproducible assessment of global RV morphology, contraction and haemodynamic performance, independently of afterload and preload. In routine clinical practice, most of these criteria are met by integrating a combination of qualitative and quantitative metrics from several imaging modalities including echocardiography, CMR, multi-detector computed tomography (MDCT) and right heart catheterisation (RHC) (Figure 4) (49).

### 4.1 Echocardiography

Echocardiography is a well established modality for the assessment of cardiac structure and function. It is widely available and versatile, making it a fundamental first line investigation for AS. Comprehensive RV evaluation requires multiple scanning planes and echocardiographic modalities, including visual 2-dimentional (2D) evaluation, M-mode, doppler, tissue doppler imaging (TDI), strain and 3-dimentional (3D) imaging.

Assessment of RV size includes measurement of RV free wall thickness, end-systolic area, end-diastolic area, basal and mid-cavitary diameters, base-to-apex length, together with the RV outflow tract diameter (50). The most common quantitative metrics of RV function on echocardiography provide an assessment of regional contraction or global systolic function. Evaluation of regional RV function involves the measurement of longitudinal RV displacement and velocity using tricuspid annular plane systolic excursion (TAPSE) and RV systolic wave velocity with TDI (RV S’). Surrogates of global RV function are fractional area change (FAC), 3D EF, global longitudinal strain (GLS) and free-wall longitudinal strain (FWLS) (51). A multi-parametric approach is adopted because no single measure is generally sufficient to describe the complex RV shape and functional changes under pathological conditions. Regional RV functional metrics have several notable limitations, including angle- or load-dependence and assessment of contractile function in a single longitudinal direction or from a lateral aspect of the basal RV free-wall. This only partially represents global function and can create potential inaccuracies in patients post-cardiac surgery or in cases of RV pacing. Global RV functional parameters depend on clear endocardial border definition, which makes them challenging to assess in some patients due to the anterior retro-sternal position of the RV. 3D echocardiography enables acquisition of full-volume datasets and closely correlates with CMR, leading to increased incorporation of this technique in the assessment of the right heart (52). RV function assessed by 3D echocardiography can circumvent most of the limitations of 2D parameters such as foreshortening and allows simultaneous characterisation of contractility of all three components of the RV (inflow, apical portion and outflow) (53). 2D speckle tracking for strain analysis can also overcome the limitations of conventional echocardiography by being less load/angle-dependent and less influenced by passive tethering. This allowing accurate quantification of regional and global myocardial function, reflecting more closely RV contractility (54). Strain and strain rate represent myocardial tissue deformation and are highly correlated with myocardial contractility (55). Therefore, these metrics may reflect global RV performance more adequately than other more simple echocardiographic parameters. However, successful implementation and reproducibility of echocardiography depends on good image quality, high frame rates, regular heart rates and expertise with advanced techniques and analysis platforms. The high interobserver variability associated with some echocardiography measures calls for more robust methods using cross-sectional imaging when possible.

### 4.2 Cardiac MRI

CMR is considered the gold standard imaging modality for quantification of RV size and function (56). CMR bypasses any potential geometrical assumptions about RV shape by measuring RV volume, mass and EF directly from the end-systolic and end-diastolic images. This is performed by summing up the area measurements from RV epicardial and endocardial border tracings using the short axis stack extending from the RV base to apex. This technique has high reproducibility and incorporates information on longitudinal and circumferential contraction of the RV. Qualitative assessment of cine views can identify features characterising PH, such as RV dilatation, RV hypertrophy, IVS deviation and pulmonary artery dilatation (57). Tissue characterisation using myocardial T1 mapping and late gadolinium enhancement for extracellular volume estimation can identify areas of myocardial fibrosis related to advanced PH at the IVS and/or RV insertion points (58). RV free-wall tissue characterisation using CMR is challenging owing to its thin wall, which limits spatial resolution (59). The tracking of myocardial deformation through the cardiac cycle can quantify RV strain impairment for the early detection of RV dysfunction in PH (60). Identification of decreased right atrial strain can also reflect the transition from compensated to decompensated RV function in PH patients (61). 4D flow CMR enables accurate assessment of vascular, transvalvular or intra-cavity flow in a volume of interest (62). In the context of PH, 4D flow CMR can identify abnormal or dynamic flow patterns in the pulmonary arteries which correlate closely with mean pulmonary arterial pressure (63). Other physiological parameters can be characterised, such as the regional pulmonary artery shear stress, which is related to vascular remodeling in PH (64). In these ways, CMR has the potential to yield important prognostic information in AS patients undergoing TAVI (65). However, the main limitations of CMR include its high cost, longer scan times, increased need for patient co-operation and reduced availability compared to echocardiography or MDCT. Furthermore, although the role of CMR is acknowledged in the most recent guidelines for managing AS, CMR is not performed routinely in all potential AVR recipients and its role for improving patient selection remains ill-defined (9, 10).

### 4.3 Multi-detector computed tomography

**Figure 5:**
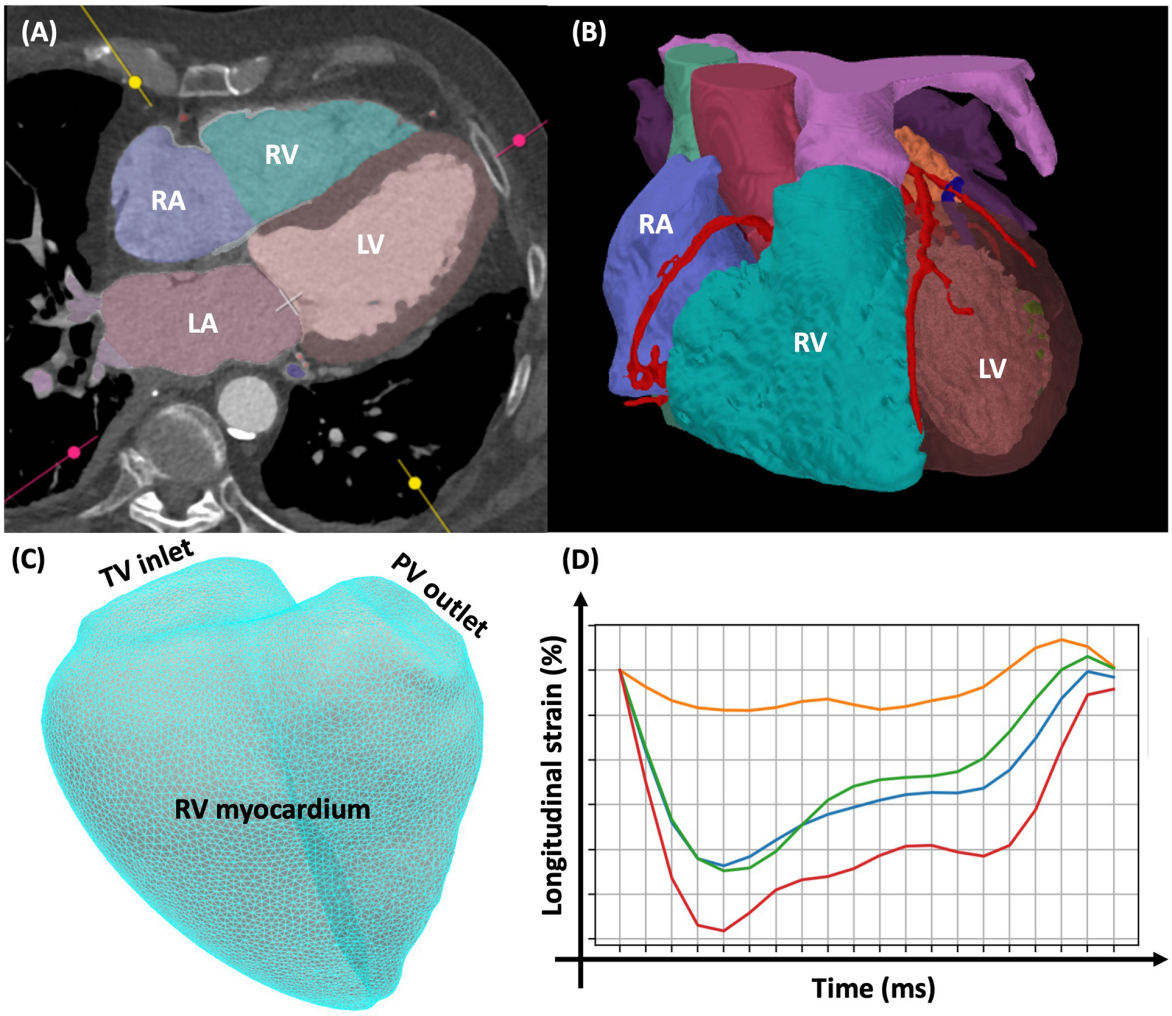
MDCT of the heart demonstrating the **(A)** anterior anatomical position of the right ventricle, **(B)** autosegmentation of the right ventricle and other cardiac chambers for volumetric analysis (heart AI, laralab, münchen, Germany), **(C)** 3D mesh of the right ventricle demonstrating the inlet, the myocardium and the outlet, **(D)** example of RV 3D longitudinal strain measurement on MDCT (cemrgApp, UK) with images reconstructed in 5% increments through the heart cycle resulting in 20 images per cardiac cycle. LA, left atrium; LV, left ventricle; RA, right atrium; RV, right ventricle; TV, tricuspid valve.

MDCT is the gold standard for the delineation of aortic root and vascular anatomy to assess TAVI feasibility (66). It is not generally considered to be a first-line technique for RV assessment because of the need for iodinated contrast and ionising radiation. However, MDCT is an appealing modality for RV assessment in potential TAVI recipients due to its routine implementation in this patient cohort. This creates an opportunity for acquiring additional useful information during the same imaging study, including cardiac chamber volumes (Figure 5). MDCT has higher spatial resolution and provides true isotropic imaging compared to CMR, which enables excellent endocardial-blood pool interface definition (67). Additional benefits for MDCT include shorter scan times, low risk of claustrophobia and no additional considerations needed for patients with implanted cardiac devices. Advances in MDCT acquisition protocols have offered the potential to improve image quality and expand the utility of MDCT for accurate assessment of RV dimensions and function. Retrospective ECG-gated acquisitions and reconstructions with thin slice thickness (<1.5 mm) for at least 10 phases of the cardiac cycle (0%–90% of R-R interval) enable full volumetric assessment throughout the cardiac cycle (68). As a result, assessment of RV function using MDCT compares favourably to CMR as the gold standard, making it a valid alternative (69). To reduce the radiation dose, tube-current modulation and low-dose scanning with model based iterative reconstruction can be implemented (70). Improvements in auto-segmentation and dynamic tracking of the RV on MDCT has played an important role in accurate RV function assessment (71). Interest is growing in the application of feature-tracking on MDCT, which allows quantification of myocardial deformation and strain, which are less dependent on loading conditions than EF (72). Statistical modelling of RV shape and image-based computational fluid dynamics may allow the assessment of early remodeling features to predict clinical outcomes (73). Further studies are needed to understand the clinical and prognostic relevance of this information in patients being considered for TAVI.

### 4.4 Invasive haemodynamic assessment of right ventricular contractility and afterload

RHC using the fluid-filled Swan-Ganz catheter allows assessment of RV preload, afterload and function by direct measurement of right cardio-pulmonary pressures (74). At present, the guidelines do not recommend routine RHC in all AS patients who are being evaluated for AVR, with indications reserved to selected patients in whom significant PH is suspected based on echocardiography screening (9, 10). This approach can be used to measure CO using several techniques including thermodilution, the direct Fick or indirect Fick (75). Other important RV functional parameters can be derived from a combination of CO, cardiac index, heart rate and the recorded pressures (Table 1). Elevated right atrial pressure represents an increase in central venous pressure and could also be indicative of RV dysfunction (76).

**Table 1:** Haemodynamic variables calculated from right heart catheterization.

Variable	Calculation method
Cardiac output (CO) using Fick equation	VO2/(Ca—Cv)
Pulmonary vascular resistance (PVR)	(mPAP—PCWP)/CO
Total pulmonary resistance (TPR)	mPAP/CO
Transpulmonary pressure gradient (TPG)	mPAP—PCWP
Diastolic pressure gradient (DPG)	dPAP—PCWP
Stroke volume (SV)	CO/HR
Cardiac index (CI)	CO/BSA
Stroke volume index (SVi)	CI/HR
PA compliance (PAC)	SV/(PASP—PADP)
RV stroke work index (RVSWi)	[(mPAP—RAP) × CI × 0.0136]/HR
PA pulsatility index (PAPi)	(PASP—PADP)/RAP

BSA, body surface area; Ca, arterial oxygen content = systemic oxygen saturation (SaO2,%) × haemoglobin (g/dl) × 1.34/100; Cv, mixed venous oxygen content = mixed venous saturation (SvO2,%) × haemoglobin (g/dl) × 1.34/100; CO, cardiac output; dPAP, diastolic pulmonary artery pressure; mPAP, mean pulmonary artery pressure; PA, pulmonary artery; PADP, pulmonary artery diastolic pressure; PASP, pulmonary artery systolic pressure; PCWP, pulmonary capillary wedge pressure; PVR, pulmonary vascular resistance; RAP, right atrial pressure; VO2, oxygen consumption.).

**Figure 6:**
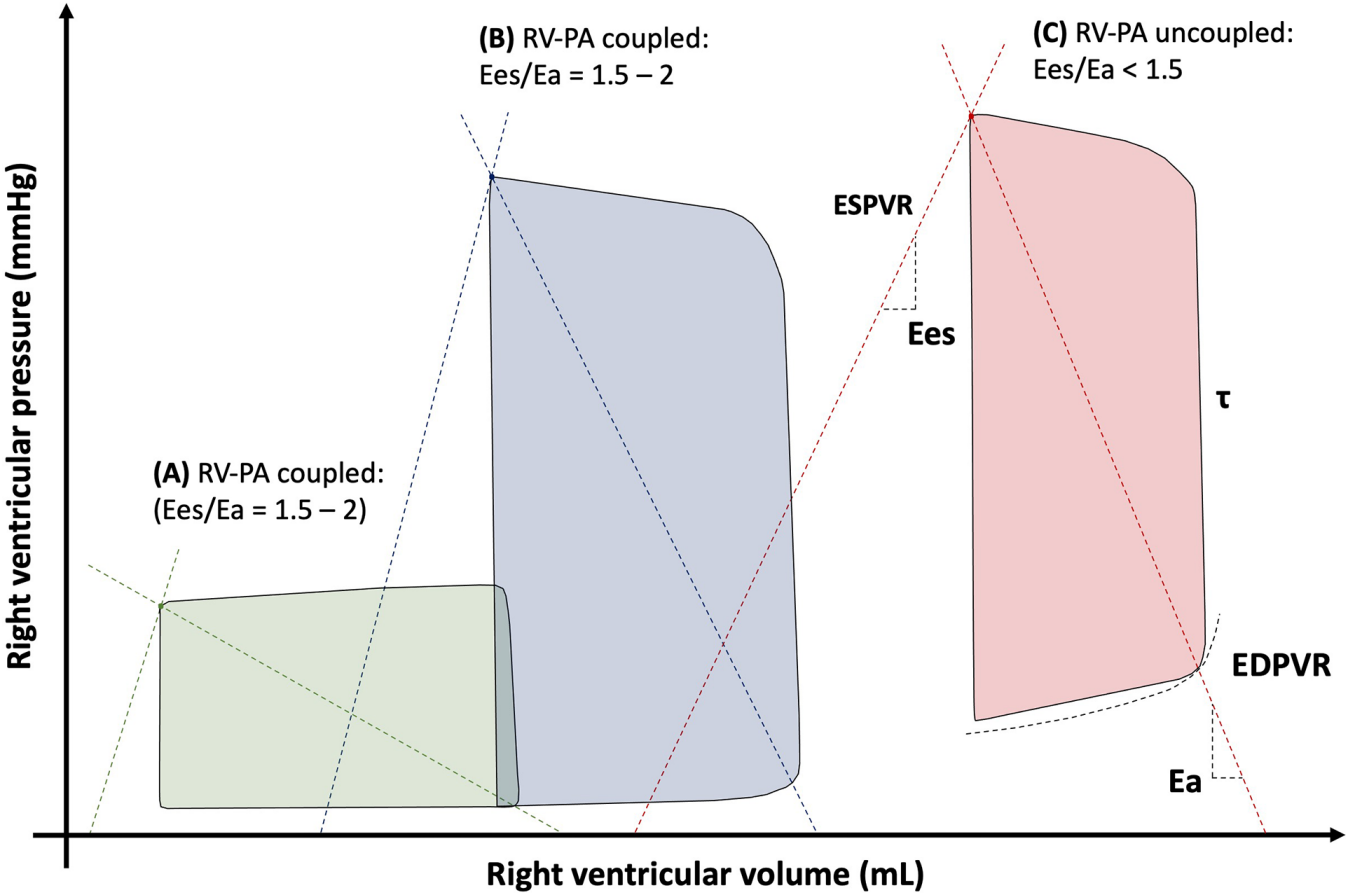
Right ventricular pressure-volume loops in **(A)** normal conditions, **(B)** pulmonary hypertension and **(C)** right ventricular failure. Ea, arterial elastance; Ees, end-systolic elastance; EDPVR, end-diastolic pressure volume relationship; ESPVR, end-systolic pressure volume relationship; *τ*, time constant of ventricular relaxation.

It should be noted that the RV metrics derived from the RHC are limited in that they provide a pressure-centric perspective of global RV function, which is determined by the intrinsic ventricular characteristics and interactions with loading conditions. The intrinsic RV contractility relates to myocardial tissue shortening forces and can be impacted positively or negatively by processes such as compensatory hypertrophy or diffuse fibrosis. Assessment of intrinsic RV contractility cannot be achieved using standard RHC alone. The gold standard method for characterising the RV systolic and diastolic properties in a load-independent fashion requires the use of specific high-fidelity electrical conductance catheters for RV pressure-volume loop (PVL) analysis (Figure 6) (77). The boundaries of the PVL are defined by the end-systolic pressure volume relationship (ESPVR) and end-diastolic pressure volume relationship (EDPVR). These relationships can be characterised using either the multi- or single-beat acquisition techniques. The multi-beat method requires a series of PVLs to be recorded under different loading conditions, whereas the single-beat method is less technically challenging and uses extrapolation to define the systolic and diastolic relationships (78). Measurements of PVL area, width and height represents RV stroke work, RV SV and PASP respectively. RV contractility is described by end-systolic elastance (Ees), which is calculated from the gradient of ESPVR. Assessment of the RV diastolic function is made by calculating end-diastolic elastance (Eed) from the EDPVR.

A full description of the cardiopulmonary circulation also requires the assessment of RV afterload, which consists of two components, namely a steady and a pulsatile load. PVR is a representation of the steady load and accounts for approximately 75% of RV afterload, whereas pulmonary artery compliance (PAC) is the description of the pulsatile load (79). The PVR and PAC is calculated from the RHC measurements, whereas total RV afterload incorporating both components is derived from PVLs and represented by effective arterial elastance (Ea) (80). Quantification of RV performance using RHC is sensitive and accurate. However, this method is invasive, technically demanding, time consuming, expensive and unpractical at bedside, limiting its routine clinically applicability to AS patients.

## 5 Assessment of right ventricular-pulmonary artery coupling

Examination of RV function and pulmonary circulation as a combined unit involves the characterisation of two components: RV contractility and pulmonary artery afterload. Several methods for RV-PA coupling assessment have been employed, including invasive and non-invasive approaches (Table 2). Using PVL analysis, the gold standard metric to express the relationship between the load-independent measures of RV contractility and total pulmonary afterload (RV-PA coupling) is the ratio between Ees and Ea (Ees/Ea). Normal coupling is maintained when the transfer of energy from the RV to the pulmonary circulation remains efficient, with Ees/Ea in the normal range between 1.5 and 2 (81).

**Table 2:** Strengths and weaknesses of invasive and non-invasive methods for measuring RV-PA coupling.

Method	Parameter	Strengths	Weaknesses
Invasive	Ees/Ea	- Gold standard - Accurate and sensitive	- Technically challenging - Specialist training and equipment - Expensive - Bedside applicability limited
Non-invasive	TAPSE/PASP	- Easy and fast - Reproducible - Less reliant on image quality	- Angle and load dependent - PASP may be under-estimated with significant TR - TAPSE reflects only longitudinal function of basal lateral segment
	S’/RVSP	- Easy and fast - Reproducible - Less reliant on image quality - S’ less dependent on afterload than TAPSE	- Angle and load dependent - S’ reflects only longitudinal function of basal lateral segment
	FAC/PASP	- Easy and fast - Reflects longitudinal and radial function - Angle independent	- Good image quality needed for clear endocardial border definition - Load dependent
	RV 3D EF/PASP	- Independent of geometric assumptions - Extensively validated against cardiac MRI	- Good image quality needed for clear endocardial border definition - Low temporal resolution on echocardiography - Specialist training and equipment
	RVLS/RVSP	- Less angle and load dependent - Less affected by RV geometry - Reproducible	- Good image quality needed for clear endocardial border definition - Specialist training and equipment - Out of plane motion of speckles
	SV/ESV	- SV calculated using 3D echocardiography is independent of geometric assumptions	- May under-estimate true RV-PA coupling

Ea, arterial elastance; Ees, end-systolic elastance; EF, ejection fraction; ESV, end-systolic volume; FAC, fractional area change; PASP, pulmonary artery systolic pressure; RVLS, right ventricular longitudinal strain; RVSP, right ventricular systolic pressure; SV, stroke volume; TR, tricuspid regurgitation; 3D, 3-dimentional.

To overcome the need for invasive techniques, there has been increased interest in other more clinically accessible alternatives to measuring RV-PA coupling. Several approaches have been proposed using predominantly echocardiography and CMR that incorporate surrogate measures of RV contractility and afterload (82). In a validation study of 52 patients with PH, echocardiographic surrogates were strongly correlated with invasive assessment of RV-PA coupling, with TAPSE/PASP ratio emerging as an independent predictor of Ees/Ea [Multivariate odds ratio (OR), 18.6; 95% CI, 0.8–96.1] (83). In receiver operating characteristic (ROC) analysis, a TAPSE/PASP cut-off of 0.31 mm/mmHg (sensitivity: 87.5% and specificity: 75.9%) discriminated RV-PA uncoupling defined as Ees/Ea <0.805. The significant correlation between TAPSE/PASP and Ees/Ea (*r* = 0.71) was confirmed in another study of 74 patients with heart failure with reduced EF and secondary PH (84). In a further study of 29 patients with idiopathic PH, both RV-FWLS/PASP and RV-GLS/PASP showed moderate but significant correlations with Ees/Ea (*r* = 0.443 and *r* = 0.529, respectively), demonstrating validity and applicability of these easily obtainable surrogates of RV-PA coupling (85). Alternative volume-based RV-PA coupling metrics have been proposed in patients where there is insufficient TR on echocardiography to estimate PASP. Ees is measured invasively as end-systolic pressure/end-systolic volume (ESP/ESV) and Ea is measured as ESP/SV, allowing the RV-PA coupling to be simplified as SV/ESV. In paediatric PH patients, SV/ESV ratio evaluated by CMR showed a significant correlation with Ees/Ea (*r* = 0.79) measured using RHC (86). The 3D echocardiography-derived SV/ESV ratio also correlates strongly with the reference RHC measurements (*r* = 0.826) (87). It should be noted that although simple and effective, these non-invasive ways of measuring RV-PA coupling are subject to the limitations of their respective imaging modalities, with the preferred first-line measure and uncoupling threshold still to be determined.

## 6 Impact of right ventricular dysfunction on clinical outcomes after AVR

Assessment of RV function can offer important clinical perspectives and prognostic information in AS. In patients with at least moderate AS under regular surveillance, the presence of RV dysfunction is a major and independent marker of poor survival [Hazard ratio (HR), 1.55; 95% CI, 1.21–1.97] (88). A growing number of studies have shown that RV dysfunction on echocardiography has significant implications on clinical outcomes in patients with severe AS undergoing AVR. In cardiac surgery patients, baseline RV dysfunction is a well-recognised predictor of post-operative decompensated heart failure (55, 89). In a study of 539 patients receiving mostly SAVR, RV dysfunction before surgery was an independent predictor of all mortality at the median follow up of 4.4 years (HR, 0.94; CI, 0.92–0.96) (90). The presence of pre-operative RV dysfunction, defined as FAC < 35%, was the strongest predictor of all-cause and cardiovascular mortality at 3 years (HR, 4.80; CI, 2.40–9.40 and HR, 14.70; CI, 6.26–31.96, respectively) in a study of 400 patients undergoing mainly SAVR (91). Similar results have been demonstrated in a more recent study of 269 patients treated predominantly with SAVR, where abnormal baseline RV function was related to increased 30-day mortality (OR, 3.50; CI, 1.10–11.1) and post-operative multisystem failure/shock (92). These adverse outcomes may be attributable to several surgery-related factors that can exacerbate the risk of post-operative RV failure in patients with initial RV dysfunction. This is related to the adverse effects of cardiopulmonary bypass on inflammatory and coagulation pathways, suboptimal cardioprotection, loss of atrioventricular synchrony, myocardial ischaemia resulting from thoracotomy and pericardiotomy and increased pulmonary artery pressures after reduced pulmonary perfusion. A meta-analysis demonstrated that compared to SAVR, TAVI is considered the preferred intervention in patients with significant AS and RV systolic impairment (93).

The acute post-surgical stressors are not relevant to most TAVI recipients and as a result, the prognostic effect of baseline RV impairment has received considerable attention in this field. The results have been summarised in a meta-analysis, which demonstrated that baseline RV dysfunction is an independent predictor of all-cause mortality 1 year after TAVI (HR, 1.31; 95% CI, 1.1–1.55) (94). In another meta-analysis, RV systolic dysfunction was associated with a 78% relative risk increase of all-cause death at 1 year after TAVI [Risk ratio (RR), 1.78; 95% CI, 1.37–2.31] (95). More recent investigations of RV function on TAVI-planning MDCT showed that increased RV volume (>120 ml/m^2^) or RV impairment, defined as RVEF < 50% or RV-GLS > −11.4%, increase the risk of post-procedural all-cause mortality and the composite outcome of death or heart failure hospitalisation 2–3 fold (96–98). Similar adverse prognostic consequence were observed in CMR studies with impaired RV-GLS > −22.0% before TAVI, validating the potential of MDCT to assess RV function in this patient cohort (99). This also illustrates the importance of assessing both RV volume and contractility indices for risk stratification in AS patients. Inclusion of these parameters in future prospectively validated clinical risk scores may have a role in improving risk assessment and patient selection for AVR.

Recently, there has been increased interest in creating a staging classification for AS based on the degree of extra-valvular myocardial remodeling (100). This approach provides a more comprehensive and patient-specific way of stratifying patients by considering downstream functional and anatomical cardiac consequences of severe AS beyond the aortic valve. According to the proposed model, patients with severe AS can be classified into 4 stages: stage 0—no extra-valvular damage, stage 1—LV damage, stage 2—left atrial/mitral valve damage, stage 3—pulmonary vasculature/tricuspid valve damage and stage 4—RV damage. This method of staging severe AS yields distinct prognostic trajectories when applied to TAVI or SAVR patients, with worst clinical outcomes in more advanced stages of cardiac remodeling, mainly RV overload or dysfunction (101, 102). Compared with Stages 0–1, Stage 4, Stage 3, and Stage 2 confer a 4.5-fold, 3-fold, and 1.5-fold increased risk of all-cause mortality and a 13-fold, 8-fold, and 4-fold increased risk of cardiovascular mortality, respectively (103). The application of this system has been validated in multiple cohorts of AS patients and applied to several modalities for categorising cardiac remodiling, including echocardiography, RHC and MDCT (104–106). The extent of cardiac damage is also associated with lower health status assessed by the Kansas City Cardiomyopathy Questionnaire Overall Score (KCCQ-OS) both at baseline (Stage 4 KCCQ score 47 vs. Stage 0 KCCQ score 66) and at 1 year after AVR (Stage 4 KCCQ score 79 vs. Stage 0 KCCQ score 88) (107). Overall, the growing body of evidence suggests that AS needs to be considered in the context of cardiac remodeling sequalae instead of a singular entity affecting the aortic valve. This classification method has important clinical implications for risk stratification, as more sensitive identification of patients prior to end-stage cardiac damage with RV dysfunction may improve outcomes by refining the timing of intervention. Additionally, recognition of advanced stages of cardiac damage may improve the prediction of expected treatment outcomes and modulate subsequent follow-up with “secondary prevention” strategies.

In patients suitable for both surgical and transcatheter treatment options for AS, it is currently uncertain whether TAVI is preferable to SAVR in patients with baseline RV dysfunction. Analyses of the PARTNER IIA trial showed that RV function deteriorated 4-fold more frequently after SAVR compared to TAVI, which was also associated with a 2-fold higher mortality risk (108). These hypothesis generating findings suggest that the presence of RV dysfunction may favour the selection for TAVI rather than SAVR. In relation to TAVI, which may be technically feasible even in the context of high/prohibitive surgical risk, old age, advanced frailty or multiple co-morbidities, it remains to be established if there is a threshold of cardiac injury related to AS above which the risk of intervention exceeds that of conservative management. It is estimated that around 50% of patients with baseline RV dysfunction do not improve RV systolic function after TAVI, despite a significant decrease in PH in the majority of patients (101, 108–111). Importantly, this is associated with a gradient of risk, with reduced cardiovascular death in patients showing recovery of RV dysfunction (HR, 2.16; 95% CI, 1.16–4.02) compared to patients with persistent RV dysfunction (HR, 8.74; 95% CI, 5.33 14.3) (112). At present, there is insufficient data to provide insights into the factors determining irreversible RV dysfunction. The fact that changes in RV function are not perfectly aligned with PASP likely reflects the multifactorial mechanisms of RV impairment in this patient population. The possibility of persistent RV dysfunction underlines the importance of considering RV function as one of the criteria for early referral and intervention.

## 7 Impact of right ventricular-pulmonary artery uncoupling on clinical outcomes after AVR

The non-invasive indices of RV-PA coupling have been increasingly investigated for their role in predicting clinical outcomes in AVR patients. In the large study of 570 low-risk patients in the PARTNER (Placement of Aortic Transcatheter Valve) 3 trial, baseline RV-PA uncoupling defined as TAPSE/PASP ratio ≤0.55 mm/mmHg was an independent predictor of all-cause mortality and rehospitalisation at 2 years after TAVI and SAVR (HR, 1.92; 95% CI, 1.04–3.57) (113). The addition of TAPSE/PASP ratio to the predictive model including age, STS score and LVEF significantly improved the prediction of adverse clinical events in 34%–52% of patients. In a study of 457 severe AS patients who underwent TAVI, TAPSE/PASP <0.29 mm/mmHg was an independent predictor of all-cause mortality (HR, 2.21; 95% CI, 1.07–4.57) after adjustment for potential confounders (114). The authors identified that this ratio could risk stratify patients in a dose-response manner, with worse uncoupling associated with the highest mortality. In 56 patients with heart failure due to severe AS undergoing TAVI, pre-procedural RV-PA coupling assessed using TAPSE/PASP (HR, 4.97; 95% CI, 5.42–21.99) and RV-GLS/PASP (HR, 2.33; 95% CI, 3.96–12.99) could predict death and HF hospitalisation better than other individual RV echocardiographic parameters (115). Further evidence from a study of 377 TAVI patients demonstrated that RV-PA uncoupling defined as TAPSE/PASP <0.36 mm/mmHg was independently associated with a more than 2-fold higher risk of 6-month mortality (HR, 2.66; 95% CI, 1.04–6.80) (116). TAPSE/PASP had better performance in predicting 6-month death after TAVI than TAPSE and PASP alone and was independent of the Society of Thoracic Surgeons (STS) risk score. The impact of other non-invasive surrogate markers of RV-PA coupling on clinical outcomes after TAVI are being increasingly investigated. Impaired baseline RV-FWLS/PASP ratio <0.63% /mmHg is an independent predictor of mortality (HR, 5.97; CI, 1.44–24.8) and of the composite endpoint of death and rehospitalisation (HR, 4.14; CI, 1.37–12.53) after TAVI (117). Collectively these results provide strong support for a more systematic and meticulous assessment of the right-sided cardio-pulmonary unit for enhanced risk assessment before AVR. Few studies have examined the clinical significance of RV-PA coupling evolution after AVR, which may further improve patient risk stratification by identifying non-responders to treatment. Emerging evidence demonstrates that persistent or new-onset RV-PA uncoupling is an independent predictor of mortality at 4 years after TAVI (HR, 1.39; CI, 1.01–1.92 and HR, 2.14; CI, 1.31–3.48, respectively), whereas normalisation of RV-PA coupling is related to better outcomes (118). Further research is needed to systematically evaluate the parameters associated with the lack of improvement or deterioration of RV-PA coupling after TAVI, which may identify patients who could benefit from closer follow-up and tailored therapeutic optimisation.

## 8 Future directions

Severe AS should be considered as the disease of the whole heart, with alterations affecting the RV structure and function characterising an advanced stage. Recent innovations in imaging techniques have created new opportunities to examine pertinent anatomical and functional changes effecting the RV in AS. Due to the inherent complexity of RV structure and physiology as well as the limitations of different imaging modalities, a quantitative multi-parametric approach is recommended. This requires skilled imaging operators as the reliability of the comprehensive assessment can vary depending on individual expertise and experience. One disadvantage of this approach is the potential for discrepant conclusions from different parameters. As a result, further research is needed to identify an accepted uniform algorithm for non-invasive assessment of the RV in the context of pre-procedural risk stratification. Detailed evaluation of subtle and sub-clinical RV changes using global and regional myocardial strain analysis could have an important prognostic role as a sensitive measure of myocardial dysfunction (119). Although still in the investigative phase, laboratory biomarkers may also have a role in screening for early RV dysfunction, with suppressor of tumorgenicity 2 (ST2), soluble ST2 (sST2), galectin-3 (Gal-3), heart-type fatty acid-binding protein, growth differentiation factor 15 (GDF-15) and neutrophil gelatinase-associated lipocalin (N-GAL) currently being studied specifically in reference to RV dysfunction (120, 121). Furthermore, there continues to be an important unmet need for a robust mortality prediction model in SAVR and TAVI patients (18). Evaluation of the RV has traditionally been poorly represented by the risk models and should be integrated in future scoring systems for a more precise and individual risk assessment. In order to successfully implement this in practice, it is of paramount importance to perform early and accurate systematic evaluation of RV size, shape and function in all AS patients using the appropriate imaging modalities available. Application of machine learning and artificial intelligence-based algorithms could offer rapid, accurate and automated means of performing RV measurements to achieve large sample sizes required for these studies (122).

Despite the increased awareness that RV dysfunction has a central role in identifying patients at increased risk of adverse outcomes after AVR, several areas of uncertainty persist. One of the main clinical questions arises in relation to the timing of intervention in significant AS with evidence of RV dysfunction. The optimal treatment of these patients remains controversial, since RV damage can persist even after successful AVR but some patients may potentially benefit from early intervention when irreversible remodeling has not yet occurred, especially since RV dysfunction can improve immediately after AVR (123). Factors predicting reverse remodeling and improvement in RV contractility and vascular coupling remain to be established, with further research on the optimal timing of intervention in this cohort being of crucial importance. It remains unknown if early signs of extra-valvular myocardial remodeling, including RV dysfunction, could be important for identifying higher risk asymptomatic patients with moderate or severe AS who may benefit from pre-emptive AVR. Several ongoing trials in severe asymptomatic AS (EVoLVeD—NCT03094143; EASY-AS—NCT04204915) and moderate AS with evidence of cardiac remodeling (PROGRESS—NCT04889872; EXPAND TAVR II—NCT05149755; TAVR-UNLOAD—NCT02661451) will provide useful insights to address this gap in the evidence. Further work is also needed to enhance our understanding of the complex haemodynamic mechanisms and molecular pathways governing RV remodeling, pulmonary vascular biology and interventricular interactions in AS. This may help to identify novel therapeutic targets and better stratify the patients at risk of developing RV-PA uncoupling, RV dysfunction and ultimately RV failure. It will be important to establish specific cut-off values for non-invasive surrogates of RV-PA coupling for successful implementation of these metrics in clinical practice. Data surrounding the impact of RV dysfunction and RV-PA uncoupling on exercise tolerance, quality of life and symptom recovery also merits further investigation. Given that MDCT is the gold-standard for assessing TAVI feasibility, the added value of this imaging modality for assessing the right heart and predicting clinical outcomes in AS requires further clarification.

## 9 Conclusions

There is a growing appreciation for the importance of RV function in patients with AS. The increased focus is driven by the mounting evidence that demonstrates worse clinical outcomes in patients with significant AS and concomitant RV impairment. Despite the clear challenges posed by the complex RV structure and physiology, the emerging evidence from different imaging modalities indicates the potential of RV evaluation to guide risk stratification and the optimal timing of intervention in patients with AS. Further work is required to integrate quantitative measures of RV function and its coupling to the pulmonary circulation into cardiovascular outcomes registries to produce improved risk stratification tools in order to facilitate appropriate patient selection and clinical decision-making before AVR.
